# Microcirculation-targeted resuscitation in septic shock: can complex problems have simple answers?

**DOI:** 10.1186/s13613-020-00796-z

**Published:** 2021-01-06

**Authors:** Jérémie Joffre, Matthieu Legrand

**Affiliations:** 1 Department of Anesthesia and Perioperative Care, Division of Critical Care Medicine, UCSF School of Medicine, University of California, 500 Parnassus Av, San Francisco, CA 94143 USA; 2 INI-CRCT Network, Nancy, France

Septic shock (SS) is a condition whereby the circulation cannot meet the tissue’s metabolic demand and/or when cellular metabolism is impaired. Microcirculatory anomalies correlate with organ failure severity and are predictive of in-ICU mortality [1], whereas systemic hemodynamic parameters failed to do so under septic conditions. Therefore, it is now considered that critically ill patients' resuscitation needs to restore sufficient tissue perfusion and oxygen delivery for cellular metabolism to prevent organ failure.

In this issue of the journal, Castro et al. [2] report a randomized clinical trial comparing CRT vs. lactate-targeted fluid resuscitation protocol. Among 42 patients, they observed that a fluid resuscitation strategy based on achieving an index finger CRT > 3 s during the first 24hours achieves more often the predefined perfusion target compared to a strategy aiming to reduce lactate below < 2 mmol/l or a decrease > 20% every 2 h. The two strategies were not statistically different for markers of tissue hypoxia (lactate pyruvate ratio and index of anaerobic CO2 generation), microcirculatory flow parameters (sublingual SDF, plasma disappearance rate of indocyanine green, and muscular StO2), and 28 days mortality. However, the CRT-based strategy trends to reduce the fluids administered and fluid balance during the first 24 h. Therefore, the authors conclude that stopping fluid administration when CRT is > 3 s is safe and could limit fluid overload.

Early correction of macro-hemodynamic parameters is a no-brainer in circulatory shock. However, early goal-directed therapy based on macrocirculatory parameters, such as higher MAP [3], CVP, SvO2 [4], or cardiac index [5], have failed to improve SS outcome. Under perfused microcirculation may persist despite early restored macrohemodynamics. Thus, targeting the microcirculation is a logical goal to obtain adequate resuscitation and avoid both over- and under-resuscitation.

Recently, the ANDROMEDA-SHOCK trial and its post hoc analysis [6] suggested that a microcirculation-guided strategy based on CRT might limit organ failure and lower mortality compared to a lactate-targeted one. In line with these results, Castro et al. study supports CRT’s use, a free and “easy to use- easy to learn” bedsides microcirculation assessment tool, to guide early fluid management in SS. However, the need for a comprehensive and efficient strategy in sepsis shock resuscitation should not overshadow the complexity of the microvascular alterations observed in SS. One might also consider that “*a CRT- based fluid protocol is a tailored strategy allowing restoration of microcirculatory flow and tissue oxygenation*,” a gross overreaching statement. Indeed, the so-called “sepsis-induced endotheliopathy,” primary substratum of microcirculatory failure, is a multi-faceted syndrome involving several distinct physiological processes [7]. For example, it seems conceptually flawed to think that immuno-thrombosis, alteration of vasoreactivity, or oxidative stress can all be reversed by a fluid challenge. Besides, alterations in the microvascular endothelium are often associated with capillary leak syndrome, in which case the fluid challenge can worsen capillary leakage, increase interstitial pressure, and ultimately tissue ischemia. While fluid loading can improve microcirculatory blood flow in early sepsis, it is obvious that the correction of microcirculatory abnormalities cannot solely rely on volemia or increase of cardiac output. This could also explain the so-called “incoherence between macro and microcirculation” observed at some point in SS patients. Moreover, the multiplicity of microvascular perfusion indices, oxygenation parameters, and hemodynamic variables, their multiple determinants, and their variations sometimes dissociated during resuscitation and sepsis course, make the dynamic integration of these parameters in real time difficult for the clinician.

Another point to consider is the lactate-guided resuscitation in the control group. It stands on the assumption that elevated blood lactate reflects organ hypoperfusion with inadequate oxygen delivery and subsequent anaerobic lactatogenesis. Nevertheless, the reasoning that hyperlactatemia must be treated by fluid resuscitation—in order to increase the cardiac output and oxygen delivery––and intensified until the blood lactate level has normalized is highly arguable. Indeed, much evidence has shown that stress hyperlactatemia is due to both an adaptive switch in cell metabolism toward increased aerobic lactate production and adrenergic stimulation [8]. The kinetic of plasma lactate and intermittent dosing further complexify its use to guide treatments. Also, if the lactate level is undoubtedly a marker of disease severity, titrate the hemodynamic management to a lactate level (or clearance) might be considered biologically inappropriate and lead to fluid overload [9].

What can we conclude from the study of Castro et al.? Probably that administrating fluids based on lactate levels can lead to excessive fluid administration, and such a strategy should be reconsidered. Then that a CRT-based strategy seems to be a useful and straightforward tool to refine fluid resuscitation efficacy assessment and ultimately improve global tissue perfusion. What is next? We should now better understand the best resuscitation strategies in septic shock, including timing and strategies for fluid administration and vasopressors but also the role of more specific treatment targeting the microcirculation, such as ilomedine. Such works are underway and results eagerly awaited [10].

## Data Availability

Not applicable.

## References

[1] Legrand M, De Backer D, Depret F, Ait-Oufella H (2019). Recruiting the microcirculation in septic shock. Ann Intensive Care.

[2] Castro R, Kattan E, Ferri G, Pairumani R, Valenzuela ED, Alegria L (2020). Effects of capillary refill time-vs. lactate-targeted fluid resuscitation on regional, microcirculatory and hypoxia-related perfusion parameters in septic shock: a randomized controlled trial. Ann Intensive Care.

[3] Asfar P, Meziani F, Hamel JF, Grelon F, Megarbane B, Anguel N (2014). High versus low blood-pressure target in patients with septic shock. N Engl J Med.

[4] Peake SL, Delaney A, Bailey M, Bellomo R, ARISE Investigators, ANZICS Clinical Trials Group (2014). Goal-directed resuscitation for patients with early septic shock. N Engl J Med..

[5] Gordon AC, Perkins GD, Singer M, McAuley DF, Orme RM, Santhakumaran S (2016). Levosimendan for the prevention of acute organ dysfunction in sepsis. N Engl J Med.

[6] Hernandez G, Ospina-Tascon GA, Damiani LP, Estenssoro E, Dubin A, Hurtado J (2019). Effect of a resuscitation strategy targeting peripheral perfusion status vs serum lactate levels on 28-day mortality among patients with septic shock: the ANDROMEDA-SHOCK randomized clinical trial. JAMA.

[7] Joffre J, Hellman J, Ince C, Ait-Oufella H (2020). Endothelial responses in sepsis. Am J Respir Crit Care Med.

[8] Garcia-Alvarez M, Marik P, Bellomo R (2014). Stress hyperlactataemia: present understanding and controversy. Lancet Diabetes Endocrinol.

[9] Marik PE (2019). Lactate guided resuscitation-nothing is more dangerous than conscientious foolishness. J Thorac Dis.

[10] Legrand M, Oufella HA, De Backer D, Duranteau J, Leone M, Levy B (2020). The I-MICRO trial, Ilomedin for treatment of septic shock with persistent microperfusion defects: a double-blind, randomized controlled trial-study protocol for a randomized controlled trial. Trials.

